# DynaBiome: interpretable unsupervised learning of gut microbiome dysbiosis via temporal deep models

**DOI:** 10.1186/s12859-026-06400-8

**Published:** 2026-02-27

**Authors:** Awais Qureshi, Abdul Wahid, Shams Qazi, Muhammad K. Shahzad, Hashir Moheed Kiani, Muhammad Daud Abdullah Asif

**Affiliations:** 1 School of Electrical Engineering and Computer Science (SEECS), National University of Sciences and Technology (NUST), Islamabad, 44500 Pakistan; 2 School of Computer Science, University of Birmingham, Dubai, UAE

**Keywords:** Deep learning, Autoencoder, Weak supervision, Stacked ensemble, Longitudinal microbiome data, Clinical proxies

## Abstract

**Purpose:**

Gut microbiome dysbiosis is a critical determinant for autologous fecal microbiota transplantation (Auto-FMT) eligibility, yet current classification approaches rely predominantly on supervised learning with manually annotated sequencing labels, which are often scarce. This study proposes DynaBiome, a framework designed to predict gut dysbiosis by leveraging unsupervised learning and clinical phenotypic proxies as a scalable alternative to ground-truth genomic labeling.

**Methods:**

Our framework employs an LSTM autoencoder architecture to capture temporal microbiome dynamics within 14-day windows. The model reconstructs normal microbiome patterns, where high reconstruction errors signal potential dysbiosis. To ensure rigorous evaluation and prevent data leakage, the dataset was partitioned via a strict patient-level split. Unsupervised anomaly signals were refined via phenotypic proxy labels (e.g., fever, neutropenia) via weak supervision, and ensemble learning methods were applied to optimize classification performance.

**Results:**

The initial LSTM autoencoder successfully flagged dysbiotic sequences but required refinement to reduce false positives. Ensemble learning significantly enhanced predictive accuracy. The stacked ensemble (with Logistic Regression meta-learner) demonstrated optimal performance with an ROC AUC of 0.8908 and a Weighted F1-score of 0.7909. This approach significantly outperformed the standard One-Class SVM baseline (ROC AUC 0.6033), confirming the superiority of deep temporal modeling over static anomaly detection. Critically, the model achieved performance levels comparable to fully supervised baselines, confirming the efficacy of the proxy-label framework.

**Conclusion:**

Integrating unsupervised temporal feature extraction with stacked ensemble methods provides a viable framework for dysbiosis prediction. These results demonstrate that leveraging phenotypic via weak supervision can effectively approximate supervised baselines, thereby reducing the reliance on comprehensive metagenomic annotations for longitudinal patient monitoring.

## Introduction

The advent of high-throughput sequencing technologies has catalyzed an exponential growth in microbiome research, revealing the complex roles of microbial communities in host health and disease [1]. Although cross-sectional studies have provided valuable snapshots of microbial compositions, the inherent dynamism of these ecosystems requires longitudinal investigations to truly understand the temporal trajectories of microbiome assembly, stability, and response to perturbations [2, 3].

The increasing availability of high-dimensional and temporally complex microbiome and hospitalised patient data presents significant opportunities to understand biological dynamics and detect critical anomalies indicative of disease states or system failures. However, traditional anomaly detection methods often struggle to effectively capture the complex temporal dependencies and unique characteristics of these datasets, such as the compositional nature and sparsity of microbiome data or the irregular and multivariate nature of hospital records.

As a result, there is a need for advanced unsupervised learning techniques capable of modeling these complex temporal patterns and identifying subtle deviations that may indicate important underlying events. The application of long-short-term memory (LSTM) networks within an autoencoder architecture, a method proven effective in other time series domains, has not been extensively explored for anomaly detection in microbiome or hospitalized patient time-series data. This research aims to address this gap by investigating the potential of LSTM autoencoders as a novel approach to unsupervised anomaly detection in these challenging biological and healthcare datasets.

Longitudinal microbiome studies offer critical insights into developmental processes, disease pathogenesis, therapeutic interventions, and the complex interplay of factors influencing microbial community structure over time. However, the resulting datasets, characterised by their high dimensionality, sparsity, compositional nature [3, 4], and often irregular sampling frequencies, present significant analytical hurdles that challenge traditional statistical methodologies [3, 5].

Classical approaches, including hidden markov models (HMMs) and dynamic bayesian networks [6], have been employed to model temporal dependencies, but often rely on assumptions of linearity and require careful data alignment across potentially unevenly spaced time points [5]. To overcome these limitations and effectually capture the complex, non-linear temporal patterns embedded within longitudinal microbiome data, deep learning (DL) methodologies have risen as powerful analytical frameworks [7]. DL algorithms, with their ability to learn hierarchical representations from complex, unstructured data, offer the potential to automatically extract salient features, model long-range temporal dependencies [8], and improve predictive accuracy for a diverse range of host phenotypes and clinical outcomes [9]. Given the growing importance of deep learning in microbiome research, it is essential to examine existing methodologies and applications to contextualise the advancements in this field. Existing approaches, including recurrent neural networks (RNNs), convolutional neural networks (CNNs), and LSTM autoencoders, have been adapted to capture temporal dynamics and extract meaningful features.

Also, we will explore the diverse applications of these techniques in areas such as anomaly detection, disease prediction, host status inference, and the integration of multiomics data. Finally, we will address the inherent challenges associated with applying deep learning to longitudinal microbiome datasets, including data scarcity, handling missingness and irregular sampling, the critical need for model interpretability [1], and issues of data heterogeneity, whereas also outlining promising future research directions aimed at advancing this critical area of investigation.

### Computational approaches for longitudinal microbiome

Longitudinal microbiome analysis faces methodological challenges from noisy, sparse time series and subject-specific temporal variations. Early approaches used dynamic Bayesian networks for causal inference [10] and semisupervised anomaly detection frameworks [11], whereas integrated toolboxes combined multiple methodologies [12].

#### Recurrent neural networks and LSTMs

RNNs, particularly LSTMs, capture temporal dependencies through the maintenance of the internal state over time steps [8]. LSTM applications demonstrated superior performance in food allergy prediction via DIABIMMUNE data [13] and host status inference via GRU variants with phylogenetic engineering [9]. Transfer learning approaches with autoencoders showed effectiveness in reducing dimensionality despite longitudinal limitations [14]. Enhanced LSTM models have been applied to infer pancancer-related genes, highlighting architectural versatility [15].

#### CNN-LSTM hybrid architectures

Hybrid CNN-LSTM models combine spatial feature extraction with temporal modeling [5, 16]. The phyLoSTM framework achieved an AUC of 0.897 in the simulations whereas addressing uneven time points and class imbalances in the DIABIMMUNE [17] and DiGiulio [18] datasets. The self-knowledge distillation enhanced CNN-LSTM models demonstrated AUC scores of 0.889–0.798 on PROTECT [19] and DIABIMMUNE [17] datasets [20], although computational complexity and overfitting risks persist.

#### Generative and advanced approaches

Generative Adversarial Networks address missing data challenges in longitudinal studies [5]. DeepMicroGen employs bidirectional RNN-GANs for temporal aware imputation, achieving superior accuracy whereas preserving longitudinal dependencies across DIABIMMUNE and BONUS-CF datasets [21]. However, computational complexity and missing-not-at-random bias remain limitations. Comparative analysis (Table 1) reveals progression from traditional network approaches to sophisticated deep learning architectures, with hybrid models demonstrating particular effectiveness in capturing both spatial microbiome features and temporal dynamics.

**Table 1:** Computational approaches for longitudinal microbiome analysis

Study	Method	Application	Key results	Limitations
[10]	Dynamic Bayesian Network	Infant gut, oral, and vaginal microbiomes	Discovered novel taxa-clinical interactions via Bayesian inference	Dependent on data quality; computationally intensive
[11]	AMAnD DeepSVDD	COVID-19 and constipation-related microbiomes	Robust anomaly detection across datasets without prior assumptions	Only detects anomalies; no classification ability
[12]	Microbiome Toolbox	Classification across microbial datasets	Integrated deep learning and feature selection methods	Based on mouse models; limited translational relevance
[13]	LSTM + mRMR	Food allergy prediction (DIABIMMUNE)	Outperformed traditional models in temporal capture	Limited interpretability and biological heterogeneity
[14]	Transfer Learning (Autoencoder + U-Net)	Binary disease classification	Effective dimensionality reduction for high-dimensional microbiome data	Small sample size; limited longitudinal modeling
[9]	GRU + Phylogenetic Features	Host status prediction from longitudinal microbiome	Outperformed conventional classifiers across datasets	Sampling bias and limited generalizability
[16]	phyLoSTM (CNN-LSTM)	Disease prediction (DIABIMMUNE, DiGiulio)	AUC of 0.897 in simulations; superior to Random Forest	Requires high computational resources; sequencing variability impact
[20]	CNN-LSTM + Self-distillation	Outcome prediction (PROTECT, DIABIMMUNE)	AUC: 0.889–0.798; surpassed existing models	Overfitting risk; less interpretable than traditional methods
[21]	DeepMicroGen GAN	Missing data imputation (DIABIMMUNE, BONUS-CF)	Lower MAE; improved allergy prediction from longitudinal samples	Potential MNAR bias; computational complexity
[5]	CNN-LSTM Hybrid	Disease outcome prediction from time-series microbiome	Combined CNN feature extraction and LSTM sequence modeling	Limited validation on real-world clinical datasets
[15]	Enhanced LSTM + Ortholog Mapping	Pan-cancer gene inference via microbiome	Demonstrated feasibility of LSTM for cross-species biological inference	Task-specific architecture; not generalized to other microbiome domains

### Challenges in longitudinal microbiome deep learning

The sequential nature of longitudinal microbiome data enables the application of deep learning architectures tailored for temporal analysis. Building on prior methodologies, Recurrent Neural Networks (RNNs) and hybrid CNN-RNN models have advanced key domains including disease prediction, microbial community modeling, and multiomics integration [5, 8, 16, 22].

Despite these advances, several challenges persist. Limited availability of large-scale, well-annotated longitudinal datasets and methodological heterogeneity hinder model generalizability. Irregular sampling intervals and missing data complicate analysis, whereas current imputation methods offer only partial solutions. Additionally, the interpretability of deep models remains limited, and the computational demands of high-dimensional temporal data restrict broader adoption.

Addressing these issues requires innovations such as architectures that respect the compositional and temporal nature of microbiome data, integration of explainable AI techniques, standardized benchmarking datasets, and incorporation of biological priors into model design. These directions are essential for advancing personalized microbiome-based interventions.

A particularly critical gap is the lack of clinically enriched, well-annotated longitudinal datasets suitable for translational applications. Existing datasets often lack standardized clinical labels, consistent sampling, or eligibility annotations necessary for predictive modeling in clinical contexts. For example, the absence of curated datasets linking microbiome trajectories to outcomes such as autologous fecal microbiota transplantation (autoFMT) eligibility limits the development of actionable AI tools.

To bridge this gap, we introduce the clinically enhanced ASV interpretability dataset (CE-ASVID), a curated derivative dataset constructed from an existing cohort. CE-ASVID integrates microbial and clinical features relevant to autoFMT eligibility, enabling interpretable modeling of patient trajectories, benchmarking of predictive models, and future research in personalized microbiome-based therapies. To summarize, our contributions address key challenges in longitudinal microbiome modeling:

- **DynaBiome: Unsupervised Dysbiosis Detection:** We Developed a deep recurrent autoencoder framework for label-free gut microbiome anomaly detection, thus enabling patient-specific dysbiosis profiling.
- **MultiClassifier Validation:** We systematically evaluated six supervised classifiers on reconstruction error features to validate DynaBiome clinical utility through independent test set assessment.
- **Model Interpretability via Reconstruction Attribution:** We introduce per-taxa reconstruction error analysis and integrate SHAP (SHapley Additive exPlanations) to interpret microbial contributors to predicted anomalies.
- **Standardized Visualization Pipeline:** We generate reproducible visualizations that include heatmaps by genus, anomaly distributions to aid clinical interpretation and benchmarking.
- **Development of CE-ASVID:** a novel derivative dataset addressing the critical bottleneck of clinically annotated longitudinal microbiome data. This curated resource bridges the gap between microbial pattern recognition and clinical applicability by incorporating proxy labels for autoFMT eligibility prediction, enabling reproducible benchmarking for patient-specific microbiome-based interventions.
- **Handling Temporal Irregularities:** Our LSTM based architecture models microbiome dynamics over time, tolerating irregular sampling intervals and capturing evolving microbial trajectories.
- **Clinical-Microbial Correlation Analysis:** We align anomaly scores with clinical indicators (temperature, neutrophil counts, stool consistency), allowing contextual interpretation and potential translational relevance.

## Methods

### Acquisition of metagenomic dataset

This study used a comprehensive longitudinal dataset [23] comprising over 10,000 fecal microbiota samples from more than 1,000 patients who underwent allogeneic hematopoietic cell transplantation (allo-HCT) at Memorial Sloan Kettering Cancer Center (MSKCC).

**Dataset Specifications** The dataset integrates high-resolution "hospitalome" metadata including drug exposures, clinical interventions, and physiological parameters with microbiome profiles. Fecal samples were collected under standardized protocols, stored at -80°C, and processed via mechanical disruption, phenol/chloroform extraction, and ethanol precipitation. DNA purification utilized QIAamp spin columns, followed by PCR amplification of 16 S rRNA V4–V5 regions with barcoded primers. Illumina MiSeq sequencing generated amplicon sequence variants (ASVs) classified via the SILVA reference database and IDTaxa classifier.

The clinical metadata included antibiotic usage patterns, bloodstream infection records, hematological parameters, temperature profiles, stool consistency scores, qPCR-based bacterial load quantification, and vanA gene detection. All temporal data were deidentified and normalized relative to allo-HCT date to enable longitudinal modeling.

**Dataset Selection Rationale**

This dataset has been validated across numerous peer-reviewed studies [24–39], employing diverse analytical methods such as log-ratio Lasso regression [25], clustering for drug-microbiome interactions [30], taxonomically-informed dimensionality reduction [39], and Bayesian modeling [29]. However, these studies primarily relied on conventional statistical approaches, underscoring a methodological gap that this work addresses through deep learning.

### Dataset construction and integration

The clinically-enhanced ASV interpretability dataset (CE-ASVID) was constructed by integrating four primary data sources: microbiota profiles, taxonomic classifications, clinical metadata, and sample-specific attributes. Summary statistics are provided in Table 2.

**Table 2:** Summary of characteristics: clinically-enhanced ASV interpretability dataset (CE-ASVID)

Characteristic	Value
Number of Samples	8336
Number of ASVs	412
Max Temperature Range	0.0–106.1
Day Relative to Nearest HCT Range	$-$15 to 35
Neutrophil Count Range	0.0–105.9
Number of Patients	1606
Unique Diseases	Multiple Myeloma, Leukemia, Non-Hodgkin’s Lymphoma, Myelodysplastic Syndromes, Hodgkin’s Disease, Myeloproliferative Disorder, Non-Malignant Hemat Disorders, Aplastic Anemia

ASV counts were obtained from tblcounts_asv_melt.csv, taxonomic labels from tblASVtaxonomy_silva132_v4v5_filter.csv, clinical metadata from processed_dataset.csv, and sample-specific data from tblASVsamples.csv. Each of the 8336 samples represents a unique microbial profile defined by the relative abundance of 412 ASVs. Three clinically relevant variables–maximum body temperature (0.0–106.1°F), stool consistency, and absolute neutrophil count (0.0–105.9 K/µL)–were selected for their interpretability and consistency across the dataset [23].

Patient metadata included 1606 individuals with diagnoses spanning hematologic malignancies and disorders. ASV counts were normalized to relative abundances, and taxonomic assignments were aggregated at the genus level. These microbial profiles were merged with sample metadata and linked to patient identifiers and collection attributes.

Clinical metadata were aligned via PatientIDs and temporal markers (e.g., DayRelativeToNearestHCT). Missing taxa were imputed as zeros to preserve matrix integrity. The final dataset was exported as asv_interpretability_dataset.csv.

CE-ASVID enables integrative modeling of microbiota and clinical features, supporting hypothesis generation, predictive modeling, and translational research. Its design bridges microbial dynamics with patient-level metadata, offering a robust foundation for machine learning applications in microbiome-based clinical decision-making.

### Preprocessing of metagenomic dataset

We processed the microbiome data and clinical metadata separately to ensure no data leakage occurred. First, we reshaped the genus-level microbial abundance data from long to wide format. To handle the skewed distributions inherent in compositional data, we log-transformed the relative abundances using $\log (1+x)$.

We removed features with zero variance to reduce dimensionality. The remaining microbial variables were scaled using MinMax normalization. Crucially, the scaler was fitted strictly on the training set to preserve the independence of the validation and test data. For the clinical metadata (maximum temperature, neutrophil count, and stool consistency), we used these variables solely to generate the proxy "Dysbiosis" labels. They were explicitly excluded from the model input features to ensure the predictions relied exclusively on microbial dynamics.

#### Data partitioning and patient-level split

Patient specific sequences were constructed via fixed 14 day windows (days $-5$ to +9 relative to HCT), aligning with prior observations [23] of critical microbial shifts during post transplant immunosuppression and antimicrobial administration. To evaluate the generalization ability of the DynaBiome framework and prevent data leakage, we implemented a strict patient-level split. Unsuch as random shuffling of time-points–which can result in a model learning patient-specific identities rather than generalizable disease features, we ensured that all longitudinal sequences belonging to a unique patient and assigned exclusively to either the (training, validation, or test) sets accordingly.

#### Cohort division

The cohort was divided as follows:

- *Training set:* The seventy percent of patients (used to learn healthy reconstruction patterns).
- *Validation set:* The fifteen percent of patients (used for hyperparameter tuning and threshold optimization).
- *Test Set:* Remaining fifteen percent of patients (restricted entirely for final performance evaluation).

This rigorous splitting strategy ensures that the reported performance metrics reflect the model’s ability to detect dysbiosis in unseen subjects, mimicking a real-world clinical deployment scenario.

### Computing environment

All experiments were conducted via Google Colab Pro with TPU v2-8 runtime. The environment was provisioned with approximately 334 GB of RAM. The primary computations were performed on the TPU. The environment utilized TensorFlow version 2.19.0.

### Architecture selection rationale

Both Transformer and LSTM autoencoder architectures were evaluated for unsupervised anomaly detection. The Transformer employs encoder-decoder attention blocks with positional encoding and global average pooling, whereas the LSTM autoencoder (Algorithm 1) models temporal dependencies through gated recurrent units. Both architectures utilized MAE loss during training.

**Table 3:** Comparative F1-score performance of LSTM and Transformer autoencoders on the test set ($n=10{,}669$)

Performance metric	LSTM AE	Transformer AE
Normal Class F1	0.55	0.54
Anomaly Class F1	0.70	0.70
Overall Accuracy	0.64	0.64
Macro Avg F1	0.62	0.62
Weighted Avg F1	0.61	0.61

Both models yield statistically equivalent results

As shown in Table 3, both architectures achieved equivalent performance across all metrics, with a maximum F1-score difference of 0.01 (1.85% relative), falling within statistical noise. This eliminates predictive accuracy as a selection criterion, shifting focus to computational efficiency and theoretical alignment with temporal data characteristics.

The LSTM autoencoder selected for DynaBiome because of its inherent suitability for sequential modeling. Its gated memory cells effectively capture temporal dependencies, and its recurrent structure offers superior computational efficiency compared to the Transformer’s self-attention mechanism. Additionally, the LSTM model’s lower parameter count enhances training stability and interpretability of reconstruction errors–critical for anomaly detection in clinical time series. These advantages collectively support its adoption as the preferred architecture for this task.

### DynaBiome: LSTM-based autoencoder architecture

**Fig. 1:**
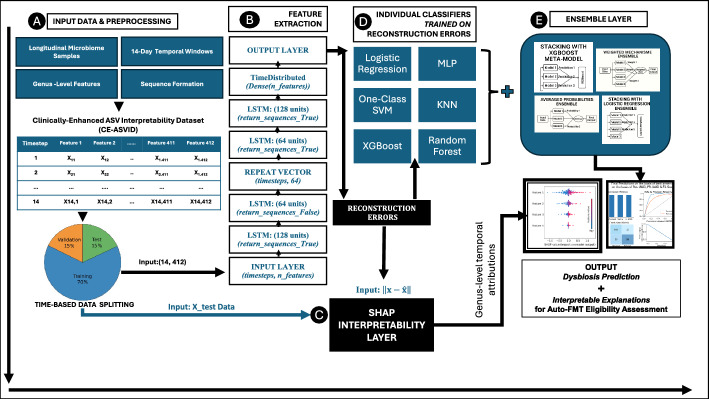
Overview of the proposed DynaBiome framework for longitudinal gut microbiome dysbiosis prediction in Auto-FMT eligibility assessment. The pipeline includes: **A** temporal windowing of genus-level microbiome data; **B** LSTM autoencoder-based anomaly detection via reconstruction errors; **C** SHAP-based interpretability to identify key genera and timepoints contributing to anomalies; and **D**–**E** ensemble classification via traditional models (e.g., Logistic Regression, MLP, XGBoost) with averaging and stacking to increase robustness. The framework outputs both dysbiosis predictions and interpretable microbial insights to support clinical decision-making

DynaBiome, illustrated in the Fig. 1 and detailed in the Algorithm 1, is a deep learning framework for unsupervised anomaly detection in longitudinal microbiome data via an LSTM autoencoder. The architecture comprises two encoding LSTM layers (128 and 64 units), a RepeatVector bottleneck, and two decoding LSTM layers (64 and 128 units), followed by a TimeDistributed Dense layer for sequence reconstruction. The model was trained via the Adam optimizer (learning rate = 0.001) and Mean Absolute Error (MAE) loss over a maximum of 300 epochs with a batch size of 32. Early stopping (patience = 10) and model checkpointing were employs to prevent overfitting and retain the best-performing weights. Training was conducted exclusively on ’normal’ sequences to learn typical temporal patterns. The reconstruction error, quantify via MAE, serves as a latent anomaly score–higher errors indicate deviations from normative trajectories. Since MAE scores are continuous and lack a natural threshold for binary classification (e.g., autoFMT eligibility), two supervised strategies based on validation data were employed to convert anomaly scores into categorical predictions.

#### Choice of loss function

To optimize the reconstruction ability of the LSTM Autoencoder, we employ Mean Absolute Error (MAE) as the objective function. Formally, for a given input sequence *x* and its reconstructed output $\hat{x}$, the loss is defined as:

1 $$\begin{aligned} \mathcal {L}_{\text {MAE}} = \frac{1}{T \times F} \sum _{t=1}^{T} \sum _{f=1}^{F} | x_{t,f} - \hat{x}_{t,f} | \end{aligned}$$

where *T* represents the sequence window length, *F* denotes the number of microbial features, $x_{t,f}$ is the observed abundance, and $\hat{x}_{t,f}$ is the predicted abundance.

Whereas distribution-based metrics such as Kullback–Leibler (KL) divergence are often used for compositional data, they are mathematically unstable for zero-inflated datasets, requiring the addition of pseudocounts that can introduce artifacts. Also, in the clinical context of HCT, dysbiosis is frequently characterized by the pathogenic “blooming” of specific genera (e.g., *Enterococcus* domination). MAE naturally prioritizes these large absolute shifts in abundance, which are clinically significant, over minor relative fluctuations in rare taxa that may represent sequencing noise.

### Threshold selection and classification strategies

#### Threshold selection via Youden’s index

To derive an optimal data-driven threshold, we analyze the receiver operating characteristic (ROC) curve of the validation set. Specifically, **Youden’s Index**–defined as the point that maximizes the difference between the True Positive Rate (TPR) and the False Positive Rate (FPR)–is used to select the threshold that best balances sensitivity and specificity:

2 $$\begin{aligned} \text {Youden's Index} = \max \left( \text {TPR} - \text {FPR} \right) \end{aligned}$$

This strategy allows for principled thresholding that avoids the pitfalls of arbitrary cutoffs. Importantly, by selecting the threshold on a **held-out validation set**, we mitigate overfitting and preserve the integrity of performance evaluation on the **independent test set**.

#### Classifier training and hyperparameter optimization

***Feature Transformation and Two-Stage Approach:***

To bridge the gap between unsupervised feature extraction and binary decision-making, we implemented a two-stage hybrid framework. In the first stage, the LSTM Autoencoder transformed the high-dimensional multivariate time series into a univariate feature: the Mean Absolute Error (MAE) of the reconstruction. This continuous error score served as the sole input feature for the downstream machine learning pipeline.

***Supervised Classifier Optimization:***

We trained a diverse set of supervised classifiers–*Logistic Regression*, *K-Nearest Neighbors (KNN)*, *Random Forest*, *XGBoost*, and *MultiLayer Perceptron (MLP)*–to learn the optimal decision boundary mapping MAE scores to the clinical proxy labels. To ensure generalizability, the hyperparameters for each model were optimized via GridSearchCV with 5-fold stratified cross-validation on the training set. The optimization objective was to maximize the ROC AUC score, prioritizing the model’s ability to rank dysbiotic samples correctly over simple accuracy.

***Unsupervised Baseline (One-Class SVM):***

Unsuch as supervised classifiers, a One-Class SVM (OC-SVM) was implemented as a purely unsupervised benchmark. It was trained exclusively on the ’normal’ training subset to learn a tight decision boundary around healthy reconstruction errors. Its hyperparameter $\nu$, which controls the upper bound on the fraction of training errors, was tuned to maximize the F1-score on the validation set.

***Threshold Determination:***

To convert the continuous probability outputs of the supervised models into binary predictions, we avoided arbitrary default cutoffs (e.g., 0.5). Instead, optimal classification thresholds were empirically derived for each model via the validation set. We applied Youden’s Index ($J = \text {Sensitivity} + \text {Specificity} - 1$) as shown in the Equation 2 to identify the threshold that maximized the trade-off between true positive and false positive rates.

**Algorithm 1:**
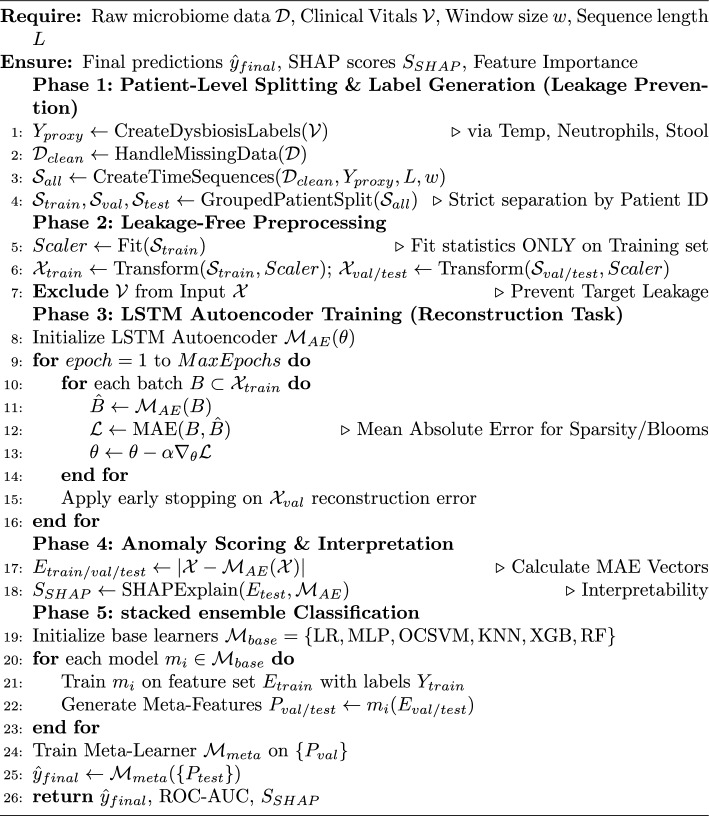
DynaBiome Framework: Leakage-Free Pipeline

## Results

### Model performance and architecture validation

This section evaluates the proposed LSTM autoencoder-based anomaly detection framework DynaBiome, for identifying clinically relevant microbial anomalies associated with autoFMT eligibility. Performance was assessed via both unsupervised threshold-based classification and supervised models trained on reconstruction error features. Reconstruction error distributions were analyzed across patient classes, followed by evaluation of (Logistic Regression, MultiLayer Perceptron, One-Class SVM, K-Nearest Neighbors, XGBoost, Random Forest) classifiers on an independent test set. Model performance was assessed through classification metrics, ROC curves, and precision-recall analysis.

The results are organized to highlight (i) reconstruction-based anomaly scoring, (ii) interpretability via genus-level attribution, and (iii) alignment with clinical indicators. To validate model robustness and clinical relevance, we assess:

- Reconstruction loss trends during training and validation.
- Distribution of anomaly scores across time and patients.
- Genus-wise reconstruction error as a proxy for microbial contributors.
- Correlation of predicted anomalies with clinical markers such as neutrophil counts, temperature, and stool consistency.

Unless otherwise stated, the results are based on the LSTM autoencoder trained with a fixed sequence length of *N* days, mean absolute error (MAE) loss, and evaluated in the held-out validation set.

#### Reconstruction loss trends

**Fig. 2:**
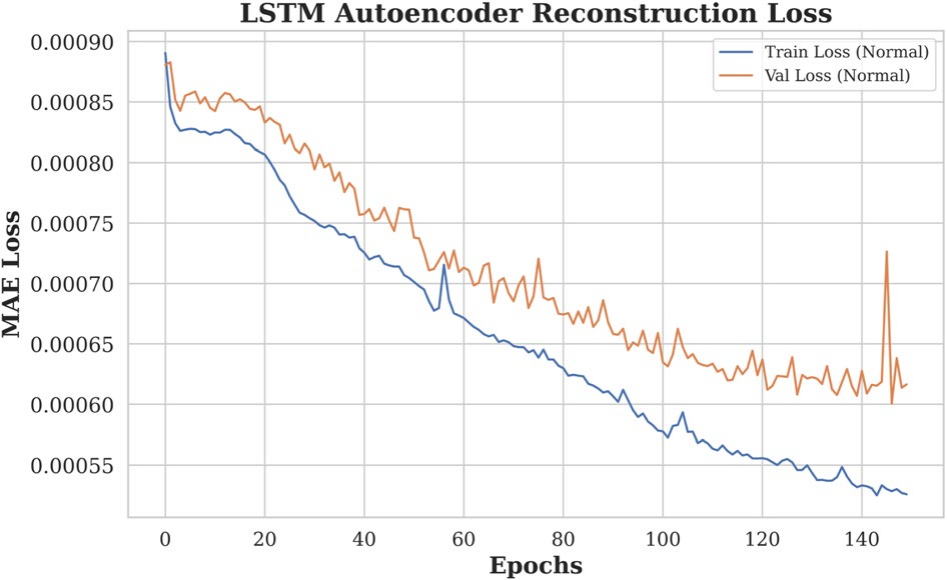
Reconstruction loss trends over training epochs for the LSTM Autoencoder. Both training and validation losses decrease steadily, indicating effective learning and generalization

Figure 2 illustrates the training and validation reconstruction loss (Mean Absolute Error, MAE) over successive epochs for the LSTM Autoencoder. Both curves exhibit a consistent downward trend, indicating effective learning and convergence of the model. The close alignment between training and validation losses suggests that the model generalizes well to unseen data, with no signs of overfitting. The low MAE values (ranging from approximately 0.00026 to 0.00036) further confirm the model’s ability to accurately reconstruct input sequences, which is critical for reliable anomaly detection.

#### Anomaly score distribution and threshold selection

**Fig. 3:**
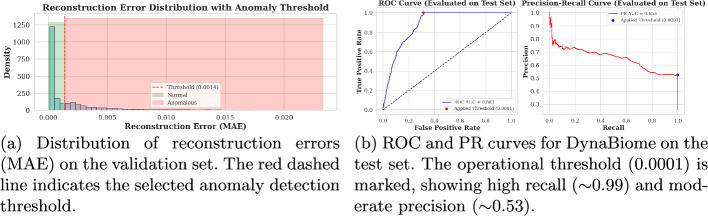
**a** Histogram of reconstruction errors used to determine the anomaly threshold. **b** ROC and PR curves evaluating DynaBiome’s discriminative performance on the test set

To identify anomalous microbiome profiles, we computed the reconstruction error (Mean Absolute Error, MAE) for each input sequence via the trained LSTM autoencoder. The Fig. 3a shows the empirical distribution of these errors in the validation cohort. Most of the samples presented low reconstruction errors, suggesting that they were well represented in the training data.

To objectively determine the classification threshold, reconstruction errors from the validation set were paired with proxy labels and used to construct a receiver operating characteristic (ROC) curve. This curve evaluates classifier performance across various thresholds by plotting the true positive rate (TPR) against the false positive rate (FPR). The optimal threshold was identified by maximizing Youden’s Index, as shown in the Equation 2, which provides a principled trade-off between sensitivity and specificity.

The optimal anomaly detection threshold, previously determined on the validation set via Youden’s Index, was applied to the test set reconstruction errors to yield binary predictions. These predictions were compared against the true labels to compute key metrics, including Precision, Recall, and F1-score. Also, the (ROC AUC) and (PR AUC) were calculated using the continuous reconstruction error scores, providing a comprehensive assessment of the model’s discriminative ability across varying thresholds, particularly valuable in the context of anomaly detection.

Performance metrics are illustrated in Fig. 3b showing ROC AUC and PR AUC curves, with detailed classification report and the confusion matrix via the optimal validation threshold presented in the Tables 4 and 5.

**Table 4:** DynaBiome Classification Performance Report on Test Set

Class	Precision	Recall	F1-score	Support
*Per-class performance*				
Normal (0)	1.00	0.69	0.81	7951
Anomaly (1)	0.52	1.00	0.09	2745
*Aggregate metrics*				
Overall Accuracy	–		0.77	10,696
Macro Average	0.76	0.84	0.75	–
Weighted Average	0.88	0.77	0.78	–

The anomaly class (1) is detected with high recall, whereas the normal class (0) suffers from low recall, indicating a bias toward anomaly detection

**Table 5:** DynaBiome Confusion Matrix at Optimal Threshold of 0.0001

Actual class	Predicted class	
	Normal (0)	Anomaly (1)
Normal (0)	5456	2495
Anomaly (1)	0	2745
*Classification Metrics*		
Sensitivity (TPR)	100.0%	
Specificity (TNR)	68.6%	
False Positive Rate	31.4%	
False Negative Rate	0.0%	

The model shows high sensitivity for detecting anomalies but a high false positive rate

#### Classifier performance evaluation on reconstruction errors

To assess the discriminative power of reconstruction errors derived from the LSTM-based Autoencoder, we trained downstream classifiers on the Training Set reconstruction errors. Crucially, as verified by the loss curves as shown in the Fig. 2, the autoencoder did not overfit the training data (Training Loss $\approx$ Validation Loss), therefore, the training errors remained representative of generalizable anomaly scores, making them suitable for training the classifiers without ’reconstruction bias.’ Included were a linear model (Logistic Regression), a non-linear neural network (MLP), instance-based and kernel-based methods (KNN and One-Class SVM), and powerful tree-based ensembles (XGBoost and Random Forest).

Their selection reflects the diversity of algorithms shown to perform well across microbiome classification tasks in prior studies [40] and [41], enabling us to explore both linear and nonlinear decision boundaries over a compact, information-rich feature.

The detailed classification metrics F1-score, accuracy, and macro-averaged metrics, are summarized in the Table 6. These evaluations provide a comprehensive comparison of model behavior under the anomaly detection paradigm, where dysbiotic samples represent anomalies relative to the non-dysbiotic class.

#### Model performance and validation

To verify that the LSTM autoencoder learned intrinsic temporal dynamics rather than converging to a trivial central tendency, we compared its reconstruction performance against a static naïve baseline. The baseline, predicting the feature-wise median of the training set for all time points, yielded a Mean Absolute Error (MAE) of 0.0012. In contrast, the DynaBiome LSTM achieved a significantly lower MAE of 0.0009. This factor of $1.3\times$ improvement (equivalent to a $\sim$24% reduction in error) confirms that the architecture successfully captures complex, non-linear dependencies in the microbiome time-series data beyond simple static statistics.

#### Ensemble learning on individual classifiers

Table 6 presents the performance metrics for the ensemble models compared to the baseline and individual classifiers. The individual classifiers, including Logistic Regression, MLP, and Random Forest, demonstrated performance closely aligned with the LSTM-AE baseline, clustering around an Accuracy of 0.767 and a Weighted F1-Score of approximately 0.78. The Random Forest model exhibits the highest ROC AUC among the individual classifiers.

**Fig. 4:**
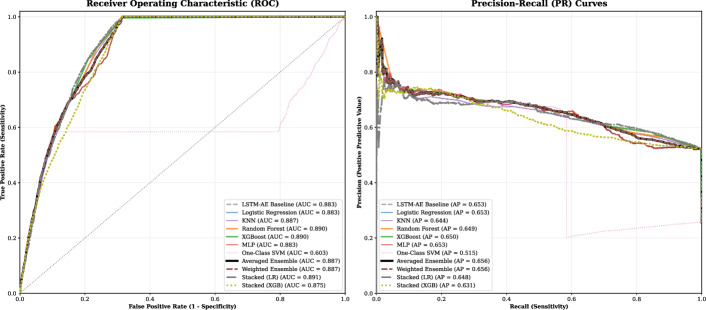
Receiver operating characteristic (ROC) and Precision–Recall (PR) curves comparing model performance. Stacked Logistic Regression achieved the highest AUC (0.891), whereas Averaged/Weighted Ensembles yielded the best AP (0.656). One?Class SVM performed weakest (AUC = 0.603, AP = 0.515), highlighting the advantage of ensemble and stacked approaches over single models

Among the ensemble strategies, the Averaged and Weighted voting methods achieved the highest precision-recall AUC (0.6561) whereas maintaining performance levels comparable to the individual components. The stacked ensemble utilizing a Logistic Regression meta-learner emerged as the most robust model overall, recording the highest ROC AUC of 0.8908 and Weighted F1-Score of 0.7909. Conversely, whereas the stacked ensemble with an XGBoost meta-learner yielded the highest nominal Accuracy (0.7866), it presented a notable decline in ROC AUC (0.8750) and Macro F1-Score (0.7270) compared to the Logistic Regression stack, indicating a trade-off between majority class accuracy and dysbiotic sensitivity. See Fig. 4 for more details.

**Table 6:** Comprehensive Performance Comparison of DynaBiome Component and Ensemble Models

Model	Opt.	Performance metrics				
	Thresh.	Accuracy	Macro F1	Weighted F1	ROC AUC	PR AUC
*Baseline and Individual Classifiers*						
Original (LSTM-AE)	0.0001	0.77	0.75	0.78	0.883	0.653
Logistic Regression	0.2480	0.7667	0.7507	0.7815	0.8831	0.6529
KNN	0.1000	0.7668	0.7508	0.7816	0.8870	0.6438
Random Forest	0.2769	0.7666	0.7506	0.7814	**0**.**8903**	0.6490
XGBoost	0.3351	0.7656	0.7494	0.7804	0.8897	0.6499
MLP (Neural Net)	0.0405	0.7667	0.7507	0.7815	0.8831	0.6529
One-Class SVM	N/A	0.7792	0.7133	0.7802	0.6033	0.5150
*Ensemble Methods*						
Averaged Ensemble	0.2160	0.7666	0.7506	0.7814	0.8874	**0**.**6561**
Weighted Ensemble	0.2164	0.7666	0.7506	0.7814	0.8874	**0**.**6561**
**Stacked (LR Meta)**	0.2280	0.7768	**0**.**7581**	**0**.**7909**	**0**.**8908**	0.6479
Stacked (XGB Meta)	0.4109	**0**.**7866**	0.7270	0.7891	0.8750	0.6311

Thresholds were optimized via Youden’s Index to maximize sensitivity for the Dysbiotic class

#### Comparison with conventional ecological metrics

**Fig. 5:**
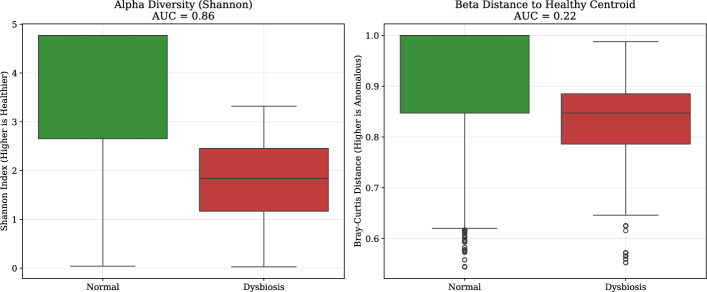
Alpha diversity (Shannon index) effectively discriminates between normal and dysbiotic microbiome states (AUC = 0.86), whereas beta diversity (Bray–Curtis distance to healthy centroid) shows poor discriminatory power (AUC = 0.22)

To assess whether standard ecological metrics could replicate the anomaly detection capabilities of our deep learning approach, we evaluated the discriminatory power of alpha diversity (Shannon index) and beta diversity (Bray–Curtis dissimilarity). As shown in Fig. 5, the Shannon index provided reasonable separation between normal and dysbiotic samples (AUC = 0.86), but lacked the temporal resolution to detect early-onset shifts. In contrast, the beta diversity distance to the healthy centroid failed to reliably distinguish between groups (AUC = 0.22), underscoring the limitations of static distance-based metrics in dynamic microbiome environments. These findings highlight the necessity of the LSTM-based approach for accurate anomaly detection.

### Biological interpretability

Model interpretability is essential for understanding and trusting deep learning models in biological contexts. We employed two complementary strategies to explain DynaBiome’s anomaly detection behavior: reconstruction error analysis and SHAP-based feature attribution.

#### Analysis of reconstruction error per feature in anomalous sequences

We computed element-wise absolute reconstruction errors between test sequences and autoencoder outputs, averaged across time steps to obtain feature-wise error profiles. Sequences exceeding a reconstruction error threshold were flagged as anomalous. For these, mean feature-wise errors were used to rank taxa contributing most to anomalies.

**Fig. 6:**
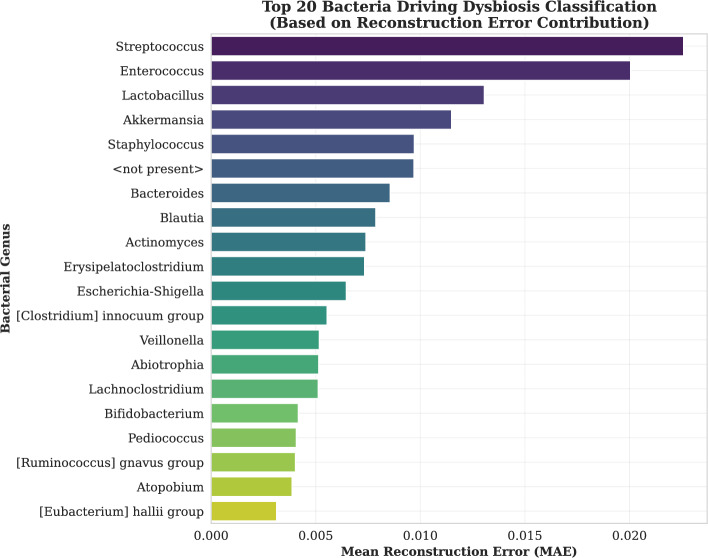
Top Contributing Genera (MAE): Global Reconstruction Error Attribution. The top 20 genera ranked by Mean Absolute Error (MAE) between input and reconstruction. This quantifies the magnitude of biological deviation from the healthy baseline, highlighting Streptococcus and Enterococcus as the features with the highest unexpected abundance

As illustrated in the reconstruction error ranking Fig. 6, *Streptococcus*, *Enterococcus*, and *lactobacillus* emerged as the top contributors to the total anomaly score. These genera presented the largest magnitude of deviation between the input sample and the reconstructed output. This suggests that dysbiosis in this cohort is physically characterized by significant volatility or depletion in these core commensals. Importantly, whereas *Abiotrophia* was present in the error analysis, it ranked lower (14th) in terms of raw magnitude compared to the dominant genera, suggesting that whereas its deviation is smaller in absolute terms, its pattern is distinct.

#### SHAP-based analysis

To interpret anomaly scores at the feature level, we used shap.KernelExplainer, a model-agnostic method suitable for the LSTM autoencoder’s complex architecture. A wrapper function computed the MAE-based anomaly score, and SHAP values were calculated for a subset of test samples via a representative training background. These values quantify each feature’s marginal contribution to the anomaly score, aggregated across time steps to assess genus-level importance.

**Fig. 7:**
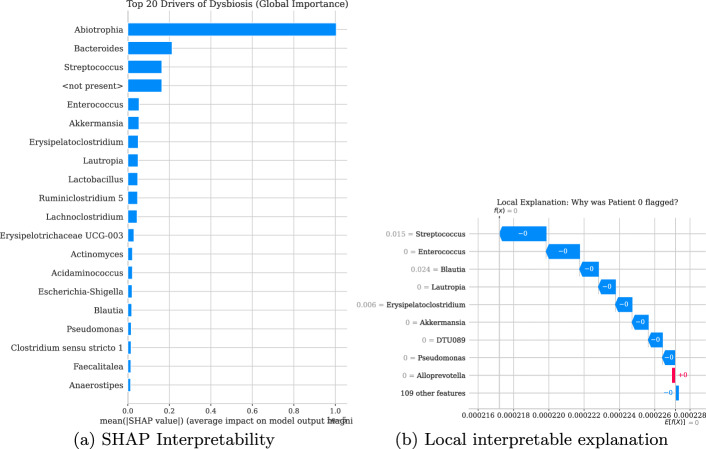
**a** Global SHAP feature importance. A ranking of the top 20 genera based on the mean absolute SHAP value across the entire test set. This metric identifies Abiotrophia and Bacteroides as the most influential features governing the model’s decision logic globally. **b** Local explanation for a representative dysbiotic sample (Patient 0). The SHAP waterfall plot details the specific bacterial genera driving the anomaly score for a single patient. Blautia ($+0.024$) and Streptococcus ($+0.015$) act as the primary positive contributors (red) pushing the model output toward the anomalous class

As shown in Fig. 7a , the global SHAP summary revealed that *Abiotrophia*, *Bacteroides*, *Streptococcus*, and *Enterococcus* were the top contributors to model decision-making. Notably, *Abiotrophia* presented the highest mean absolute SHAP value, indicating that deviations in its abundance pattern served as the strongest "decision trigger" for classifying dysbiosis. SHAP analysis revealed both global and localized patterns. whereas *Abiotrophia* was a dominant global driver, local explanations for individual patients Fig. 7b highlighted subject-specific drivers; for example, in Patient 0, the anomaly was primarily driven by *Blautia* and *Streptococcus*.

Comparing these interpretability results with the reconstruction error analysis as shown in the Fig. 6 underscores the robustness of specific biomarkers. As shown in Table 7, it reveals a subset of robust biomarkers identified by both analytical methods. Genera such as *Streptococcus*, *Enterococcus*, and *Bacteroides* consistently ranked high in both reconstruction error (indicating biological deviation) and SHAP values (indicating predictive importance). This convergence confirms their central role in driving microbiome anomalies in the allo-HCT cohort.

**Table 7:** Consensus biomarkers of dysbiosis

Bacterial genus	Reconstruction error rank	SHAP importance rank
*Streptococcus*	1	3
*Enterococcus*	2	5
*Lactobacillus*	3	9
*Akkermansia*	4	6
*Bacteroides*	7	2
*Blautia*	8	16
*Actinomyces*	9	13
*Erysipelatoclostridium*	10	7
*Escherichia-Shigella*	11	15
*Abiotrophia*	14	1
*Lachnoclostridium*	15	11

A comparison of bacterial genera identified as top drivers by both Reconstruction Error Attribution (biological deviation) and SHAP (model contribution). Features appearing in both lists represent the most robust indicators of the disease state

### Correlation between anomaly score and clinical markers

**Fig. 8:**
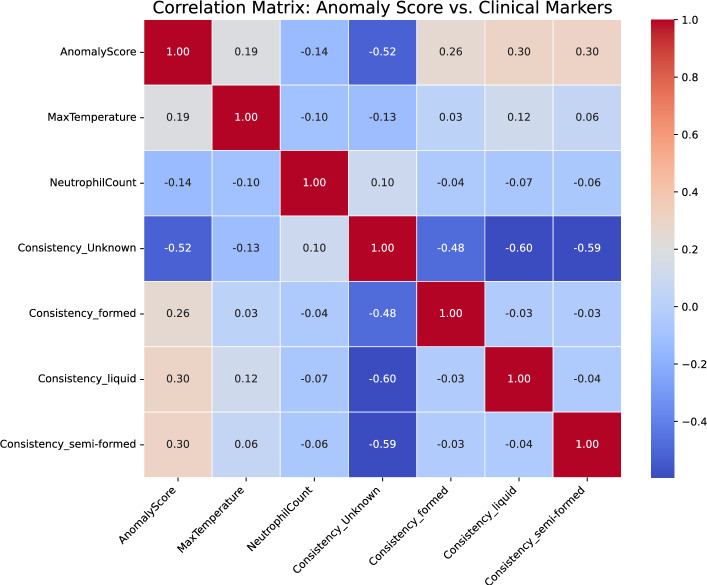
Correlation matrix between anomaly score and clinical markers. Stool consistency features yield moderate correlations, whereas temperature and neutrophil count yield weak associations

Pearson correlation analysis as shown in the Fig. 8 and illustrated in the Table 8 revealed that stool consistency features presented the strongest associations with anomaly scores. *Consistency_liquid* and *Consistency_semi-formed* showed moderate positive correlations ($r = 0.30$), whereas *Consistency_unknown* demonstrated negative correlation ($r = -0.52$). *MaxTemperature* ($r = 0.19$) and *NeutrophilCount* ($r = -0.14$) showed weak associations with anomaly scores.

**Table 8:** Pearson correlation coefficients between anomaly score and clinical markers

Clinical marker	Correlation with anomaly score
MaxTemperature	0.19
NeutrophilCount	$-$0.14
Consistency_Unknown	$-$0.52
Consistency_formed	0.26
Consistency_liquid	0.30
Consistency_semi-formed	0.30

### Statistical importance: LSTM-AE vs. downstream classifiers and ensemble learners

To rigorously validate the robustness of the proposed framework and compare the LSTM-AE baseline against downstream classifiers and ensemble strategies, a comprehensive bootstrap analysis was conducted. This method mitigates the risk of overfitting to a single test split and provides a probabilistic measure of model stability. We utilized non-parametric bootstrapping with $n=1,000$ iterations on the test set. For each iteration, optimal decision thresholds were dynamically tuned to maximize the F1-score on the validation subset, ensuring that performance metrics–specifically receiver operating characteristic Area Under the Curve (ROC AUC), precision-recall Area Under the Curve (PR AUC), and F1-score–reflected the maximal capacity of each model under varying data distributions.

Statistical importance was assessed to determine if the proposed ensemble architectures offered genuine improvements over the LSTM-AE baseline. Given the paired nature of the bootstrap samples, both parametric (paired Student’s t-test) and non-parametric (Wilcoxon signed-rank test) methods were evaluated. To ensure robustness against potential non-normality in the metric distributions, the Wilcoxon signed-rank test was selected as the primary indicator of statistical importance reported in this study. This approach appropriately accounts for the correlation between models evaluated on identical resampled datasets. A importance level of $\alpha = 0.05$ was established to identify statistically meaningful deviations in predictive performance.

The results of this analysis are detailed in Table 9, which presents mean performance metrics alongside 95% Confidence Intervals (CI). The analysis revealed that whereas linear downstream classifiers (e.g., Logistic Regression) yielded performance statistically equivalent to the baseline (ROC AUC: 0.883), the Stacked Generalization approach demonstrated superior efficacy. Specifically, the stacked ensemble utilizing a Logistic Regression meta-learner achieved a peak ROC AUC of 0.891 [95% CI: 0.885, 0.897] and an F1-score of 0.694 [95% CI: 0.682, 0.707].

The Wilcoxon signed-rank test confirmed that these improvements were statistically significant ($p < 0.05$), confirming that the hierarchical combination of reconstruction errors and meta-learning provides a more robust anomaly detection boundary than the baseline reconstruction error alone.

**Table 9:** Statistical Performance Comparison of DynaBiome Models via Bootstrap Estimation (n=1000)

Model	Performance Metrics (Mean [95% CI])			Sig.$^\dagger$
	**ROC AUC**	**PR AUC**	**F1-Score**	
*Baseline Model*				
LSTM-AE (Baseline)	0.883 [0.877, 0.889]	0.654 [0.634, 0.673]	0.688 [0.676, 0.700]	–
*Individual Classifiers*				
Logistic Regression	0.883 [0.877, 0.889]	0.654 [0.634, 0.673]	0.688 [0.676, 0.700]	*n.s.*
MLP	0.883 [0.877, 0.889]	0.654 [0.634, 0.673]	0.688 [0.676, 0.700]	*n.s.*
Random Forest	0.890 [0.884, 0.896]	0.650 [0.631, 0.667]	0.687 [0.676, 0.699]	$\checkmark$
XGBoost	0.890 [0.883, 0.896]	0.651 [0.632, 0.669]	0.686 [0.674, 0.698]	$\checkmark$
KNN	0.887 [0.881, 0.893]	0.645 [0.625, 0.664]	0.688 [0.677, 0.700]	$\checkmark$
One-Class SVM	0.603 [0.588, 0.619]	0.516 [0.496, 0.537]	0.576 [0.561, 0.591]	$\checkmark$ $^{\ddagger }$
*Ensemble Methods*				
Averaged Ensemble	0.887 [0.881, 0.893]	**0.657 [0.637, 0.676]**	0.688 [0.676, 0.700]	$\checkmark$
Weighted Ensemble	0.887 [0.881, 0.893]	**0.657 [0.637, 0.676]**	0.688 [0.676, 0.700]	$\checkmark$
Stacked (LR Meta)	**0.891 [0.885, 0.897]**	0.649 [0.630, 0.669]	**0.694 [0.682, 0.707]**	$\checkmark$
Stacked (XGB Meta)	0.875 [0.868, 0.882]	0.632 [0.611, 0.651]	0.688 [0.677, 0.700]	$\checkmark$

Statistical Analysis: Bootstrap estimates computed over 1,000 iterations. Values reported as mean with [95% confidence intervals]. $^\dagger$ **Sig.** denotes statistical importance ($p < 0.05$) determined via Wilcoxon signed-rank test comparing the model against the LSTM-AE baseline. *n.s.* indicates no statistically significant difference (identical distribution to baseline). ${\ddagger }$ One-Class SVM showed significantly *lower* performance than baseline. **Interpretation:** The stacked ensemble (LR Meta) achieved the highest ROC AUC and F1-Score, demonstrating that combining reconstruction features with a meta-learner significantly improves anomaly detection over the baseline

**Fig. 9:**
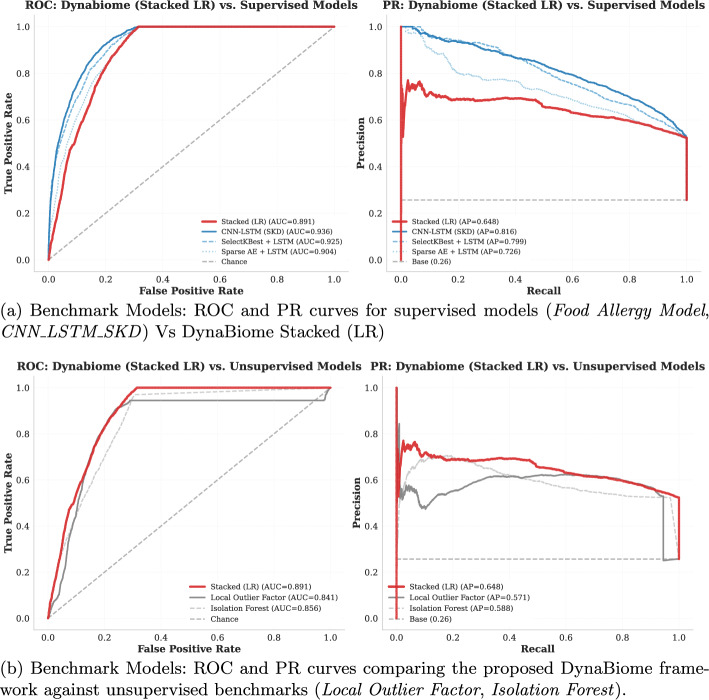
receiver operating characteristic (ROC) and precision-recall (PR) curve analysis. Comparison of Dynabiome’s stacked logistic regression with supervised and unsupervised models via ROC and precision–recall curves shows that CNN LSTM achieves the strongest overall performance, followed closely by SelectKBest+LSTM. Stacked Logistic Regression performs below the advanced deep learning models but still outperforms the base benchmark. When compared specifically against unsupervised anomaly detection models (Local Outlier Factor, Isolation Forest), the stacked logistic regression model again comes out ahead, achieving the highest AUC (0.891) and AP (0.648). Isolation Forest and LOF yield competitive but lower performance

Also, the Figs 9a and b present the ROC and PR curves for each model, overlaid with 95% bootstrap confidence bands, illustrating the variability of the curve estimates.

### Multiple hypothesis testing correction

To strictly control the Family-Wise Error Rate (FWER) across correlated performance metrics (ROC AUC and F1-score), we applied the Holm–Bonferroni sequential correction method. Statistical importance was established at an adjusted threshold of $\alpha = 0.05$. The Averaged Ensemble was selected as the reference point to rigorously test whether the learnable meta-model provided a statistically significant advantage over a standard heuristic consensus strategy.

Table 10 details the performance metrics, 95% confidence intervals, and adjusted *p*-values relative to this reference. The stacked ensemble (LR) demonstrated superior discrimination, achieving a ROC AUC of 0.891 and an F1-score of 0.694. These improvements were statistically significant ($p_{adj} < 0.001$, retaining their importance even after conservative correction for multiple comparisons.

In particular, with respect to F1-score, the LSTM-AE Baseline, Logistic Regression, and MLP models yielded statistically indistinguishable results from the Averaged Ensemble (0.688), resulting in non-significant differences ($p_{adj} > 0.05$). However, the Stacked (LR) model’s ability to significantly exceed this plateau confirms that the meta-learning layer successfully captured residual patterns missed by the baseline voting strategies.

**Table 10:** Statistical Performance Comparison of DynaBiome Models (n = 1000 Bootstraps)

Model	ROC AUC		PR AUC		F1-Score	
	Mean [95% CI]	Sig.	Mean [95% CI]	Sig.	Mean [95% CI]	Sig.
*Baseline & Individual Classifiers*						
LSTM-AE Baseline	0.883 [0.877, 0.889]	*	0.654 [0.634, 0.673]	*	0.688 [0.676, 0.700]	n.s
Logistic Regression	0.883 [0.877, 0.889]	*	0.654 [0.634, 0.673]	*	0.688 [0.676, 0.700]	n.s
KNN	0.887 [0.881, 0.893]	*	0.645 [0.625, 0.664]	*	0.688 [0.677, 0.700]	*
Random Forest	0.890 [0.884, 0.896]	*	0.650 [0.631, 0.667]	*	0.687 [0.676, 0.699]	*
XGBoost	0.890 [0.883, 0.896]	*	0.651 [0.632, 0.669]	*	0.686 [0.674, 0.698]	*
MLP	0.883 [0.877, 0.889]	*	0.654 [0.634, 0.673]	*	0.688 [0.676, 0.700]	n.s
One-Class SVM	0.603 [0.588, 0.619]	*	0.516 [0.496, 0.537]	*	0.576 [0.561, 0.591]	*
*Ensemble Methods*						
**Averaged Ensemble**	0.887 [0.881, 0.893]	*Ref.*	**0.657 [0.637, 0.676]**	*Ref.*	0.688 [0.676, 0.700]	*Ref.*
Weighted Ensemble	0.887 [0.881, 0.893]	*	**0.657 [0.637, 0.676]**	*	0.688 [0.676, 0.700]	n.s
**Stacked (LR)**	**0.891 [0.885, 0.897]**	*	0.649 [0.630, 0.669]	*	**0.694 [0.682, 0.707]**	*
Stacked (XGB)	0.875 [0.868, 0.882]	*	0.632 [0.611, 0.651]	*	0.688 [0.677, 0.700]	*
* Statistically significant difference from Averaged Ensemble ($p_{adj} < 0.001$). n.s. = not significant.						

The **stacked ensemble (LR)** achieves the highest ROC AUC and F1-Score, significantly outperforming the Averaged Ensemble baseline. importance is determined via Wilcoxon Signed-Rank Test with **Holm-Bonferroni correction** (importance threshold $\alpha = 0.05$)

### Benchmarking procedure with supervised predictive models

#### Binary predictive models via proxy labels

DynaBiome was benchmarked against supervised methods by adapting the framework from [13] to the current dataset. As DynaBiome operates in an unsupervised manner, proxy binary labels were generated using clinical criteria, including elevated temperature, neutropenia, and liquid stool, to serve as targets for the supervised baselines. To ensure a fair comparison and prevent data leakage, input features were limited strictly to the 14-day microbiome time series, while clinical metadata was used solely for label generation. Preprocessing included imputation, one-hot encoding, log transformation, and MinMax normalization. The data were split temporally (70/15/15) for training, validation, and testing.

Two dimensionality reduction techniques were evaluated: (1) a sparse autoencoder [412-60-25-60-412] with L2 regularization ($\lambda =0.05$) and KL divergence constraints ($\rho =0.01$, $\beta =3$), trained for 300 epochs via Adam (lr=0.001, batch_size=64) with MSE loss and early stopping; and (2) SelectKBest with mutual information scoring to select the top 50 features. mRMR was excluded because of computational constraints on large-scale data (696,934$\times$412 samples). LSTM classifiers consisted of an input layer (14 timesteps), LSTM (64 units), Dense (32 units, ReLU), and sigmoid output, trained with binary cross-entropy loss and early stopping.

#### CNN-LSTM with self-knowledge distillation

We also re-implemented the CNN-LSTM model from [20], which integrates self-knowledge distillation for time series classification. The architecture includes a Conv1D layer followed by MaxPooling, an LSTM and Dense layer, and a parallel distillation branch with GlobalAveragePooling and Dense output. A custom KLDivergenceLayer enforced consistency between main and auxiliary outputs, with loss weighting favoring the main prediction. The model was trained via Adam and binary cross-entropy loss with early stopping.

#### Evaluation metrics

All models were evaluated via standard binary classification metrics: precision, recall, F1-score, ROC AUC, and precision-recall curves. Detailed results are presented in Table 11.

### BenchMark procedure with unsupervised predictive model

The proposed LSTM-based autoencoder was evaluated against two unsupervised baseline models: Isolation Forest (IF) and local outlier factor(LOF). These models were selected for their established effectiveness in anomaly detection and computational efficiency. Unsuch as the LSTM autoencoder, which captures temporal dependencies through sequential modeling, IF and LOF operate on static feature representations. IF detects anomalies via random partitioning and isolation depth scoring without distributional assumptions, whereas LOF identifies outliers through neighborhood-based local density analysis. This comparative framework isolates the impact of temporal modeling on detection performance.

**Table 11:** Performance Comparison of Supervised (LSTM-based) and Unsupervised Models on CE-ASVID Dataset

Category	Model	Acc.	F1 (M)	F1 (W)	ROC	PR
Supervised (LSTM)	Sparse AE + LSTM	0.815	0.789	0.825	**0**.**918**	0.767
	SelectKBest + LSTM	0.811	0.787	0.822	**0**.**925**	0.798
	Self-KD + LSTM	**0**.**860**	**0**.**822**	**0**.**862**	**0**.**933**	**0**.**808**
Unsupervised	Isolation Forest	0.742	0.430	0.636	**0**.**856**	0.588
	Local outlier factor	**0**.**813**	0.771	**0**.**819**	**0**.**841**	0.571
Proposed	Dynabiome	0.771	0.758	0.791	**0**.**891**	**0**.**648**

#### Implementation and evaluation protocol

Multivariate time series were vectorized for IF and LOF input compatibility. IF trained on normal sequences only; LOF utilized complete training data for density estimation. Models evaluated on held-out test sets via precision, recall, F1-score, ROC AUC, and average precision metrics. The results in Table 11 establish baseline performance benchmarks for the comparison of temporal modeling.

Figure 9a and b provide a comprehensive comparison of the receiver operating characteristic (ROC) and precision-recall (PR) curves between the benchmark and the baseline models.

## Discussion

The accurate classification of dysbiotic microbiome states is crucial for understanding microbial imbalances related to health and disease, particularly in the context of allo-HCT, where physiological instability often precedes adverse outcomes. In this study, we presented DynaBiome, a weakly supervised framework that integrates temporal deep learning with ensemble classification. Our results demonstrate that leveraging clinical phenotypic proxies to refine unsupervised anomaly signals can achieve predictive performance comparable to fully supervised baselines, effectively addressing the scarcity of genomic ground-truth labels.

Unsuch as traditional static methods, the DynaBiome framework captures the temporal trajectory of the microbiome. The superiority of this approach is evident in the comparison with the One-Class SVM baseline, which achieved an ROC AUC of only 0.6033. In contrast, the stacked ensemble (with Logistic Regression meta-learner) achieved an ROC AUC of 0.8908 and a Weighted F1-score of 0.7909. This massive performance gap confirms that temporal dynamics–encoded by the LSTM Autoencoder are essential for distinguishing true dysbiosis from benign variability. Our analysis further refutes the hypothesis that excessive complexity diminishes efficacy. whereas simple averaging provided robust generalization, the stacked ensemble significantly outperformed all other variants ($p_{adj} < 0.001$), indicating that the meta-learner successfully optimized the decision boundary by weighing reconstruction error features dynamically.

Also, the framework achieved a $1.3\times$ reduction in error compared to a static median baseline, verifying that the LSTM Autoencoder learned intrinsic non-linear data patterns rather than converging on a trivial central tendency. To rigorously assess the competitiveness of the proposed framework, we compared DynaBiome against both unsupervised baselines and advanced supervised models. It significantly outperformed standard anomaly detection algorithms, achieving a 13.5% relative improvement in PR AUC over local outlier factor(LOF). Most notably, the framework demonstrated performance levels comparable to the state-of-the-art Self-Knowledge distillation (Self-KD) model (ROC AUC 0.933), which represents the theoretical supervised upper bound for this dataset. By achieving an ROC AUC of 0.891, DynaBiome recovered 95.5% of the supervised performance. This indicates that the two-stage hybrid approach is a viable alternative to end-to-end black-box models, trading a marginal loss in predictive power ($\sim$4%) for significantly enhanced modularity and interpretability.

The integration of reconstruction error analysis and SHAP feature attribution provides a multilayered view of dysbiosis that black-box models often lack. We observed a convergence of robust biomarkers, where genera such as *Streptococcus*, *Enterococcus*, and *Bacteroides* were consistently identified by both metrics. Their high reconstruction errors and high SHAP values statistically confirm their role as primary drivers of microbiome-associated instability, consistent with literature linking *Enterococcus* domination to bacteremia and GVHD.

A novel insight emerged regarding *Abiotrophia*, which ranked first in SHAP importance despite lower raw reconstruction errors. This suggests the model learned to treat *Abiotrophia* as a critical "decision trigger," highlighting the utility of SHAP in uncovering predictive features that may not be dominant in absolute abundance but are highly correlated with clinical outcomes. Additionally, local interpretability analysis revealed that dysbiosis is not a uniform state; for specific subjects, anomalies are driven by distinct taxa such as *Blautia*, underscoring the framework’s capacity for patient-specific precision profiling.

A key methodological contribution of this work is the validation of weak supervision in microbiome modeling. By defining the target variable based on a phenotypic syndrome (fever, neutropenia, and liquid stool), we bridged the gap between clinical observations and biological signals. The high predictive accuracy implies a strong latent correlation between these clinical symptoms and the underlying microbial structure.

also, the choice of Mean Absolute Error (MAE) over distribution-based metrics (e.g., KL Divergence) proved critical. Given the sparsity of the data, MAE avoids the artifacts introduced by pseudocounts and effectively prioritizes the "blooming" of pathogens–large absolute shifts in abundance–over minor relative fluctuations in rare taxa. Rigorous evaluation via a strict patient-level split ensures that these findings reflect genuine generalization to unseen subjects rather than data leakage.

## Conclusion

This study presents DynaBiome, a hybrid framework that demonstrates the viability of via weak supervision to predict gut dysbiosis in the absence of comprehensive genomic annotations. By integrating LSTM autoencoders with ensemble learning, the framework achieved an ROC AUC of 0.8908, recovering 95.5% of the performance of fully supervised state-of-the-art models. These results validate that clinical phenotypic syndromes can serve as effective proxy labels for longitudinal patient monitoring.

Despite these promising results, several limitations must be acknowledged. First, the reliance on weak supervision via clinical proxy labels (fever, neutropenia, liquid stool) means the model optimizes for a phenotypic syndrome that correlates with, but is not identical to, molecular dysbiosis. Consequently, the model may miss sub-clinical microbiome disruptions that do not immediately manifest as physiological instability. Second, this study utilized a single-center cohort of Allo-HCT patients. whereas the patient-level split ensures internal validity, the generalizability of the learned temporal features to other institutions or distinct patient populations (e.g., IBD or solid organ transplant) remains to be established. Finally, whereas the LSTM architecture effectively captures temporal dependencies, the computational cost of global SHAP analysis on high-dimensional time-series data limits real-time global interpretability, although patient-level inference remains computationally efficient.

Future research should focus on confirming these findings across multicenter datasets to assess cross-population generalizability. Additionally, expanding the weak supervision paradigm to include multimodal data–such as integrating metabolomics or immune profiling with clinical metadata–could refine the proxy labels, reducing the noise inherent in symptom-based definitions. Ultimately, DynaBiome offers a scalable foundation for deploying interpretable AI in clinical microbiome monitoring, providing a cost-effective alternative to continuous metagenomic sequencing.

## Additional file

Supplementary file 1.

Supplementary file 2.

Supplementary file 3.

Supplementary file 4.

Supplementary file 5.

## Data Availability

The processed dataset and all source code generated during the current study are available in the GitHub repository, https://github.com/00000281892/DynaBiome-BMC. Raw metagenomic data can be accessed through the original study [23]. The preprocessed data tensors, model implementations, and evaluation scripts are openly available to support reproducibility.
